# Patients' Contributions to Hospitals

**Published:** 1888-05-12

**Authors:** W. Burdett-Coutts


### Patients' Contributions to Hospitals.

By Mr. W. Burdett-Coutts, M.P.

(*Continued from p.* 81.)

### Description of the Pay System.

What is the Pay System? It may be the payment by the patient of a fixed sum for his treatment, and this is in practice the simplest plan. Probably, with regard to the out-patient department, the individual payment would be so small as hardly to permit of variation. But I am bound to say that the rigid fixed scale is not correct in theory and does not in practice go to the heart of the matter—the just relationship between eleemosynary assistance and self-help. Briefly, then, the true Pay System is that every patient should pay as much as he can towards the expenses of his treatment, and that the deficiency should be made up from the income of the hospital, whether derived from annual charitable contributions or from funded property.

### How to Treat any Surplus arising from its Adoption.

Wherever that income is more than sufficient to make up the deficiency, the surplus can go to (1) paying medical officers—this is one of those professional elements in the case which I said I should not enter upon, but I may be permitted to express a strong opinion that no general adoption of the Pay System, particularly if it includes the better class of special wards, would be just or admissible without this provision; (2) improvement of existing accommodation and comfort; (3) providing additional accommodation, by putting out other district hospitals under the management of the central institution; (4) relieving the patient of a portion of his payment. But there is another means of disposing of it, and I confess that I wish that I could look forward to the establishment of such a true confraternity of sympathy and interest amongst these institutions that this way might be preferred, viz., that those hospitals which, after the adoption of the Pay System, find themselves in possession of any considerable surplus, should extend a helping hand to their weaker brethren and apportion the whole or some part of that surplus amongst neighbouring hospitals which—and there are many of them—from want of funded property or from meagreness of annual contributions, must still, even with a rigid Pay System be placed in constant and increasing financial difficulties. We can picture the enthusiasm and the coldness with which such a proposal would be received respectively by the two parties to the contract; but viewed in the light of the identity of their objects and the general efficiency of their work, the parable of the Good Samaritan applies not less to institutions than to individuals. A system such as that which I have suggested, while it would leave to each institution an unimpaired solidarity and independence, would but serve to effectuate the common object of all such institutions, the sum of human happiness, in the utmost possible relief of human suffering. But this is a digression which it is not necessary to pursue further. It is not an integral part of the subject of the Pay System in individual hospitals. How far that system, as I have described it, obtains at present in England will appear later on. It remains to point out certain existing methods germane to it and partaking of its character.

### Existing Methods.—(1.) Governors' Letters.

I do not consider the widely prevailing system of governors' or subscribers' letters entitling to free admission and to treatment in a hospital fulfils to any appreciable extent the conditions of the Pay System. It is true that in some cases care is exercised in the disposal of these letters, but in the great majority of cases the subscriber considers that he is entitled to do what he likes with his letter; he does not examine closely into the circumstances of the patient to whom he gives it; his field, so to speak, for comparison of necessity is restricted; and it follows that when he gives his letter to some one who could afford to pay something towards his treatment—a domestic servant, for instance, in receipt of good wages—there may be dozens of poor persons outside the scope of his knowledge who have more pressing need of that free letter than the person to whom it was given, and yet have no chance of getting into communication with the governor possessed of the letter. The result is that the governor's *protegé* occupies the free bed to the exclusion of the absolutely poor. But it may be said that the governor has made his contribution on certain conditions, viz., that he should have the privilege of presenting a patient, and that we have no right to go behind that compact and inquire how he exercises that privilege, and that if we abolish this system we shall stop the flow of support to our hospitals. I do not believe this. I believe that the great mass of contributions to hospitals are given on higher grounds than those of personal privilege, that the donors in the main are animated by pure motives of charity, and that they would gladly surrender their individual rights to a system which they were satisfied would automatically secure the just and accurate distribution of the benefits of their charit where and how it was most needed.

### How to modify these.

I believe the contributing public to be directed by a spirit of justice as well as by the spirit of charity, and that any sound, intelligible combination of the two would be more acceptable to them than the almost indiscriminate indulgence of the one. Moreover, the letters ceasing to entitle to free treatment, and the Pay System being introduced, it would always be open to a governor, and it would only be fair that he should have a prior right, to present a patient and pay out of his own pocket either the whole cost or such portion of it as, under the rules of the system, he would have to pay. This method is largely adopted in cottage hospitals, and it seems to me to be more satisfactory than the "free letter" system, because it enables a governor to know what he is paying for an individual case, and what he is giving to the general fund of the hospital.

But, taking the system as it is, and supposing for the moment that every governor does carefully inquire into the cases he presents, and presents none to free treatment except the absolutely poor, no one will deny that there are a great many subscribers who never use their privilege, or, to put it more accurately, there is a large portion of the funds contributed to the hospital against which this right is never exercised at all, and that such portion falls entirely under the operation of the present conditions which I am criticising. I arrive, then, at three conclusions with regard to the existing system of free letters to subscribers—(1) that the plan does not satisfy the requirements of the Pay System; (2) that the Pay System might be so arranged as to entitle subscribers to equal privileges with better and more accurate results; (3) that the abolition or modification of the present method of free letters would not lessen charitable contributions.

### (2.) Free Letters in Return for Workmen's Subscriptions.

There is another class of free letters which, however, do answer to the Pay System, and should certainly be included in any new scheme. I refer to letters granted to bodies of workpeople in return for subscriptions raised amongst themselves, either for a particular hospital, as in the case of the fund raised for the North Staffordshire Infirmary, Hartshill, and other instances to be found in the provinces, or to form a collective fund, to be subsequently apportioned amongst several hospitals, the largest example of which is the Hospital Saturday Fund. It is true that the disposal of the letters may not involve the graduated pay principle, but we may safely trust the working people, whose money secures the letters, to see to it amongst themselves that they are given to the most deserving and necessitous cases. This practice of workmen's contributions is based on principles of self-help and a healthy recognition of responsibilities which should be in every way encouraged. I prefer the first method —that of funds raised for particular hospitals—as being more direct in its application and in its return, and as giving the subscribers a greater personal interest in some particular institution.

THEY SHOULD CARRY A SHARE OF THE HOSPITAL GOVERNMENT.

I would venture here to remark that I think that such contributions ought to entitle the contributing bodies to some share in the government of those institutions to which they contribute. I do not think that the government of hospitals should be left solely to the few people who give considerable donations or to those who have specific knowledge of hospitals, or to the medical profession itself, but that it should include representatives from all classes which contribute to its expenses. If we had representatives from bodies of work-people who contribute to the funds, and thereby obtain free admission to the hospital, we should gain what in my opinion would be a second advantage, we should have inmates and patients of the hospital represented in its government. Many would feel that they had at once a responsibility and a privilege—a stake in the welfare of the institution, extending beyond the mere time that they or their fellow-workmen were inmates of it, and involving wider considerations than the mere receipt of personal relief. I, for one, should have no fear of such an element in the government of any hospital. The people who stand all day behind counters, who work all day and sometimes all night in factories and workshops, in addition to the experience they gain in being treated, either themselves or their fellows, in a hospital, are possessed in the main of a clearness of judgment and a sense of responsibility which leaves little room for doubt as to the part they would play in hospital government.

EXAMPLES—(*a.*) SUNDERLAND.

These suggestions were made publicly now five years ago, and in connexion with this subject I would call attention to the remarkable development of a plan illustrating some of these points, under the guidance of Mr. A. W. Webster in Sunderland. I venture to send a word of greeting and congratulation to the working men of Sunderland, who, in times of great depression and want of employment, which have fallen with exceptional severity upon that town, have worked with such energy and loyalty for the institutions from which they derive benefit.

Almost every artisan and labourer in and around the town contributes his penny per week to the infirmary as regularly as he receives his wages, the penny being deducted by the employer, at the request of the workmen, and paid over to the secretary of the infirmary; many firms have a column ruled in the wages book to allow for this deduction. Each workshop or friendly society—they number about sixty—contributing £10 per annum to the funds of the institution appoints a representative who is styled a workmen's governor of the infirmary. These representatives meet quarterly, or in case of need more frequently, and are constituted the Court of Workmen's Governors, having their own chairman and officers, and conducting their proceedings according to a proper code of rules. From their body the workmen send four governors to sit on the general committee of the institution, and four to the special committee for the election of the honorary medical officers, thus maintaining the true principle of representation throughout; and, in addition to this, the Sunderland workmen also contribute to the support of the other charities in the town in the manner described by Mr. Webster :—

"Some have a special subscription now and again for a particular charity, this being in addition to the regular infirmary penny, whilst others contribute more than a penny per week, and give the extra subscription to the smaller charities. Others, again, subscribe the penny only, and some a proportionate subscription to the amount of wages earned, that is, those having under ten shillings a-week contribute a halfpenny; those above ten shillings and under thirty, give one penny; and those above thirty shillings give twopence. The workmen then decide yearly what proportion of their subscriptions shall be given to each institution."

SHIPOWNERS' SUBSCRIPTIONS.

Owners of vessels trading with the port have also voluntarily imposed a tax upon themselves of from sixpence to five shillings per voyage, according to the tonnage of the ship, towards the support of the institution where sailors, like other workmen, receive free treatment in case of sickness. The out-patient department of this hospital was turned into a provident dispensary some seven or eight years ago. The total result of this admirably-conducted experiment has been to convert the Sunderland Infirmary from a "ticket" or "letter" hospital into a free one, but a free hospital where nearly 50 per cent. of the income is derived from the patients themselves and the class to which they belong.

(*b.*) MINERS' HOSPITALS – GUISBOROUGH.

I find similar systems, with results as gratifying, existing in certain miners' hospitals in the north. For instance, at the Guisborough Miners' Accident Hospital the miners, by a contribution of one halfpenny deducted every week from wages, raise £105. Eleven subscribers, chiefly mine-owners, give £122. The income was £240, and the expenditure £200. There is a reserve fund of £447. The secretary states: "Our system works very satisfactorily. The only matter which causes any difficulty is the reception of patients who are not workmen at any of the mines, as we receive no payment (*i.e.* subscription) from them." But such cases are provided for in the rule that when any person not connected with any mine shall be taken in, "the employer shall be requested to contribute to the institution." This is, veritably, the rigid Pay System.

HAMMERWICH.

Let me take another instance—for these strong Northerners are an interesting study—the Hammerwich Cottage Hospital, of whose invigorating example the following three extracts will give a fair description :—

"The working men of the district subscribed over a-fourth of the building fund, and now their subscriptions towards the maintenance are about half the yearly cost; most of the friendly societies in the district also subscribe."

"The hospital being entirely supported by voluntary contributions, mainly from the miners of the district (and other working men), we consider patients not of that class (*i.e.* small shopkeepers, &c.) ought to pay something towards their maintenance, and charge, entirely according to their circumstances, from three shillings and sixpence to ten shillings per week, their ability to pay or otherwise being vouched for by a subscriber."

"Patients are to pay a weekly sum, dependent upon their circumstances, but in all cases the subscriber recommending the patient to be entitled to further recommend that the patient be admitted free; for this purpose the subscriptions for any body of working men will be treated as an individual subscription. The standard rate to be paid shall be seven shillings per week; such rate to be modified by executive committee if desirable.

"N.B.—To prevent any misinterpretation of this rule, it may be pointed out that it means free admission to all workmen belonging to a subscribing colliery or other works, as their aggregate subscription will cover the cost of patients at the above standard rate."

And as to the system being satisfactory, the secretary answers—

"Perfectly. We are better able to deal with the many accidents met with in the district, which is some twelve or fourteen miles from a general hospital, and serious cases of illness which it would be impossible to treat with expectation of success are frequently removed to hospital with best results."

Not Closely Applicable to London Population.

It may be urged that the methods possible to a town like Sunderland, with its numerous great workshops, and to the still more solid communities of the mining districts, might not be generally applicable to the more sporadic and disbanded elements of a London population. But where they have been really tried, they have proved successful; for instance, to the Dreadnought Seamen's Hospital, Greenwich, the ships and sailors of all nations have contributed annually £1,400, and this is on the increase. Again, in a great town like Glasgow the collections on the Clyde have amounted to from £10,000 to £15,000 annually. It is, however, to the guiding principle of the Pay System, self-help, as developed in those places, that I wish to call particular attention. It amply illustrates the spirit of sturdy self-reliance and the systematised effort which characterise the workmen of the North of England.

(3.) Free Hospitals Confined to the Absolutely Poor Coincide with Pay System.

There is one more form of the Pay System which, by an apparent paradox, does not involve any payment at all, and that is when a hospital, after careful inquiry, admits none but the absolutely necessitous. It is obvious that such a hospital, although not possessing many of the advantages which I shall claim for a combination of free and pay beds, does, by admitting to free treatment none who can afford to pay in whole or in part for themselves, answer to the economic requirements of the Pay System. This leads me to say that those hospitals, which, by the conditions of their charters or of their endowments, as in the case of the Royal Free Hospital, Gray's Inn Road, or even by the preference of their governors, remain absolutely free, would, by attaching this restriction to their admission of patients, assist rather than impair the efficiency of the Pay System.

*(To be continued.)*

